# Is global quality of life reduced before fracture in patients with low-energy wrist or hip fracture? A comparison with matched controls

**DOI:** 10.1186/1477-7525-6-90

**Published:** 2008-11-03

**Authors:** Gudrun Rohde, Glenn Haugeberg, Anne Marit Mengshoel, Torbjorn Moum, Astrid K Wahl

**Affiliations:** 1 Department of Rheumatology, Sorlandet Hospital, Kristiansand, Servicebox 416, 4604 Kristiansand, Norway; 2 Institute of Nursing and Health Sciences, Medical Faculty the University of Oslo, Pb.1153 Blindern, 0316 Oslo, Norway; 3 Dept. of Behavioural Sciences in Medicine, Medical Faculty, University of Oslo, 1111, Blindern, 0317 Oslo, Norway; 4 Centre for Shared Decision Making and Nursing Research Rikshospitalet, N-0027 Oslo, Norway

## Abstract

**Background:**

The aims of the study were (i) to examine global quality of life (GQOL) before fracture in patients with low-energy wrist or hip fracture compared with an age- and sex-matched control group, and (ii) to identify relationships between demographic variables, clinical fracture variables, and health- and global-focused quality of life (QOL) prior to fracture.

**Methods:**

Patients with a low-energy fracture of the wrist (n = 181) or hip (n = 97) aged ≥ 50 years at a regional hospital in Norway and matched controls (n = 226) were included. The participants answered retrospectively, within two weeks after the fracture, a questionnaire on their GQOL before the fracture occurred using the Quality of Life Scale (QOLS), and health-focused QOL using the Short Form-36, physical component summary, and mental component summary scales. A broad range of clinical data including bone density was also collected. ANOVA and multiple linear regression analysis were used to analyse the data.

**Results:**

Osteoporosis was identified in 59% of the hip fracture patients, 33% of the wrist fracture patients, and 16% of the controls. After adjusting GQOL scores and the three sub-dimensions for known covariates (sociodemographics, clinical fracture characteristics, and health-focused QOL), the hip patients reported significantly lower scores compared with the controls, except for the sub-dimension of personal, social, and community commitment (p = 0.096). Unadjusted and adjusted GQOL scores did not differ between the wrist fracture patients and controls. Sociodemographics (age, sex, education, marital status), clinical fracture variables (osteoporosis, falls, fracture group) and health-focused QOL explained 51.4% of the variance in the QOLS, 35.2% of the variance in relationship and marital well-being, 59.3% of the variance in health and functioning, and 24.9% of the variance of personal, social, and community commitment.

**Conclusion:**

The hip fracture patients had lower GQOL before the fracture occurred than did controls, even after adjusting for known factors such as sociodemographics, clinical variables and health-focused QOL. The findings suggest that by identifying patients with low GQOL, in addition to other known risk factors for hip fracture, may raise the probability to target preventive health care activities.

## Background

Low-energy fracture may be understood as result of a complexity of many factors related to disease, events and circumstances that may lead to injury, ultimately resulting in fracture [1-6]. Osteoporosis is a well known risk factor for low energy fractures, and Norway has a high incidence of fractures related to osteoporosis compared to the rest of the world [7-10]. Furthermore, most patients with a low-energy fracture are elderly. In Norway it is expected a growing number of elderly people in the years to come, and thereby one may expect an increasing number of low-energy fractures [11,12]. These facts highlight the need to focus on the complexity of issues related to the occurrence of low energy fractures in the elderly population.

In addition to osteoporosis, age, gender, lifestyle, falls, and concomitant medical conditions are among known risk factors for low-energy fractures [2,5,13-15]. However, also psychological, social and environmental characteristics may influence on whether or not people fall, which in turn results in fractures [16-20]. The individuals' global quality of life (GQOL), understood as satisfaction with life [19,21], may be one such factor that may add explanations to the complexity of fractures [19,20]. Research has found that GQOL is related to perceived general health, functioning, and symptom load [16,18-20,22]. Poor functioning and symptom load may result in falls, which in turn result in fractures [2,5,6,9,10,14,15,23,24]. Knowledge of GQOL prior to fracture in combination with objective factors which might be associated with the occurance of low energy fractures, might increase the possibility for health promoting activities in specific risk groups. Therefore, it is of interest to look further into the issue of GQOL prior to low-energy fractures.

Wrist and hip fractures are the most common types of low-energy fractures. The Scandinavian countries have the highest incidence of hip fracture in the world, and there is no clear explanation of the reasons for this [8,9,25,26]. Hip fracture patients are typically characterised by older age, and large complexity in their underlying conditions, co morbidities, and clinical histories prior to fracture [2,8,9,13,24,27]. When it comes to wrist fracture patients less is known about characteristics prior to the fracture. However, patients with wrist fractures are mostly elderly without severe morbidities and clinical histories [10,24,28]. In both hip and wrist fractures studies have been preformed to evaluate health – focused quality of life (QOL) issues such as function, well-being, disability and personal evaluation of health phenomena, prior to the fracture. These studies suggest that hip fracture patients have reduced health-focused quality of life even before the fracture occur [29-32]. The wrist patients have a modest decrease in health-focused quality of life within physical domain and scores in accordance with controls within mental domain assessed up to two years before the fracture [29]. However, little is known about perception of GQOL, understood as satisfaction with life, in low-energy fractures in hip and wrist. To our best knowledge no studies have been performed with this perspective in low-energy hip and wrist fracture patients.

A broader perspective on the characteristics related to the occurrence of low-energy fractures, including pre-fracture GQOL, may lead to a better understanding of the complexity of the circumstances related to low-energy fractures in wrist and hip, which in turn may leave opportunities to identify groups of individuals who might benefit from prevention efforts [18,20]. Based on this background, the aims of this study are:

(i) to examine GQOL prior to fracture in patients with low-energy wrist or hip fractures compared with an age- and sex-matched control group, and

(ii) to identify relationships between demographic variables, clinical fracture variables, health-focused QOL, and GQOL prior to fracture.

## Materials and methods

### Design

We used a comparative cross-sectional study design that included elderly patients with low-energy wrist and hip fractures and sex- and age-matched control subjects randomly selected from the general population within the study's catchments area. The patients were retrospectively asked to describe their situation before the fracture occurred within a short time span after the fracture or before being included in the study in the controls. The study was approved by the Regional Committee for Medical Research Ethics and the National Data Inspectorate.

### Patients and control subjects

#### Patients with low-energy fractures

Patients with low-energy wrist or hip fractures aged 50 years and older treated at a regional hospital in the southern part of Norway from January 2004 to December 2005 were invited to the Osteoporosis Centre for assessment of bone mineral density (BMD) and health status. The Osteoporosis Centre is organized around a fracture liaison service [33]. The fundamental principle is that nurses identify all patients treated at the hospital for low-energy fractures and invite the patients to an osteoporosis assessment. Using the risk factors identified, a physician considers the need for non-pharmacological and pharmacological actions to prevent future fractures. Before inclusion in this study, we confirmed that the fracture was not a result of high-energy trauma and was caused only by minimal trauma according to the definition of low-energy fracture [34]. We excluded patients with confusion or dementia, serious infection, tourists, patients not capable of giving informed consent, and patients not capable of speaking Norwegian.

Data was collected over two years. During this period, 324 wrist fracture patients and 456 hip fracture patients with a low-energy fracture were treated at the hospital; 249 of the patients with a wrist fracture and 307 of those with a hip fracture were examined at the Osteoporosis Centre. Sixty-eight wrist and 210 hip fracture patients were excluded (21 wrist patients and 134 hip patients) or were unwilling to participate in the study (47 wrist patients and 76 hip patients). The final study sample comprised 181 wrist fracture patients (response rate 66%) and 97 hip fracture patients (response rate 52%). Three hip fracture patients who also had a wrist fracture were counted as hip fracture patients only. All patients were examined after surgery. The median time between fracture and examination at the Osteoporosis Centre was 10 days (interquartile range; 13) for wrist fracture patients and four days (interquartile range; 2) for hip fracture patients.

Thirty of the patients with a wrist fracture and 251 of those with a hip fracture were excluded from the examination at the osteoporosis centre or from participating in the study because of dementia or because they were unable to give informed consent. Fifteen of the wrist and seven of the hip patients were tourists. Six wrist and 13 hip fracture patients were excluded due to other exclusion criteria.

Controls were identified randomly from the national registry for the catchment area and were invited to participate in the study by mail. We aimed to include one control person who was matched for age and sex for each patient. A total of 389 potential control subjects were invited to participate, of whom 226 were willing to participate (response rate of 58%). Despite several attempts, we were unable to find age- and sex-matched control subjects for some of the patients aged 75 years and older.

### Instruments

#### Demographic and clinical variables

Demographic data, BMI, whether the patients and controls exercised for at least 30 minutes three times a week (yes/no), co-morbidity, medication, smoking habits, and the number of falls before the fracture or inclusion in the control group were recorded. Falls, fracture groups or controls, and osteoporosis were regarded as clinical fracture variables in the multiple regression analyses.

#### Bone density measurements

Four trained nurses took standardized BMD measurements at lumbar spine L2–L4 and both hips using the same dual-energy X-ray absorptiometry (DXA) equipment (General Electric, Lunar Prodigy). The machine was stable over the entire measurement period. The in vivo coefficient of variation for the measurement procedure was 1.19% at lumbar spine L2–L4, 0.95% at the right total hip, and 0.89% at the left total hip. The BMD measurements were expressed as T-scores (SD) calculated on the basis of the reference value in the DXA machine provided by the manufacturer. Osteoporosis was defined as a T-score ≤ -2.5 SD according to the World Health Organization (WHO) definition for osteoporosis [34].

#### GQOL: Quality of Life Scale (QOLS)

The Quality of Life Scale (QOLS) is a 16-item, domain-specific instrument adapted by Burckhardt et al. [35] for use with chronic disease patients. In this questionnaire, GQOL is understood as a broad range of human experiences related to one's overall well-being and satisfaction [35-38]. The QOLS is a self-administered questionnaire. In our study, the patients were asked to rate their level of satisfaction with the above-mentioned dimensions at the time before the fracture. The items are rated at a 7-point satisfaction scale. For incomplete questionnaires, the missing values were replaced with the mean value of the answered questions of the respondent if 80% of the questions were completed [16].

The questionnaire is scored by adding up the items to obtain a total score from a minimum of 16 to a maximum of 112. Higher scores indicate better GQOL. Burckhardt et al. [21,39] suggested that the QOLS comprising three sub-dimensions: relationship and marital well-being (items 3, 4, 5, 6, and 14); health and functioning (items 1, 2, 11, 15, and 16); and personal, social, and community commitment (items 7, 8, 9, 10, 12, and 13) [21,38]. The three dimensions are scored by summing the scores for each item in the dimension. The questionnaire has satisfactory reliability and validity and has been tested for psychometric properties in several countries, including Norway [21,39-41]. The Cronbach's alpha in our study was 0.87 for the total score, 0.67 for the relationship and marital well-being score, 0.70 for the health and function score, and 0.76 for the personal, social, and community commitment score. The correlations between the sub-dimensions range from r = 0.54 (relationship and marital well-being, and personal, social, and community commitment) to r = 0.63 (health and function, and personal, social, and community commitment), demonstrating a moderate correlation between the dimensions [42].

#### Health-focused QOL: Short Form-36 (SF-36)

The Short Form-36 (SF-36) was used to assess health-focused QOL. The fracture patients were asked to evaluate their health status in the four weeks before the fracture and the control group in the four weeks before the BMD assessment at the Osteoporosis Centre. The Medical Outcome Study (MOS) SF-36 is a self-reported, generic health-focused QOL questionnaire. The questionnaire includes eight domains (general health, bodily pain, physical functioning, role limitations physical, mental health, vitality, social functioning, and role limitations emotional), which can be combined into a physical and mental subscale. These physical component summary (PCS) and mental component summary (MCS) scales were used in this study. The SF-36 scales were scored according to published scoring procedures, and each was expressed as a value from 0 to 100, with 100 representing excellent health [43,44]. This questionnaire has satisfactory reliability and validity. The questionnaire has been tested thoroughly for psychometric properties in several countries, including Norway [45,46]. Chronbach's alpha in our study in the eight SF-36 domains were 0.85 for bodily pain, 0.57 for general health, 0.91 for physical function, 0.91 for role limitation physical, 0.82 for mental health, 0.87 for vitality, 0.85 for social function and 0.78 for role limitation emotional.

### Statistical analysis

Statistical analysis was carried out using the Statistical Package for Social Sciences (SPSS) for Windows (version 14.0). Demographic and clinical variables were compared between groups using the chi-square test for categorical variables and ANOVA with Bonferroni adjustment for continuous variables.

Multiple linear regression analysis (procedure GLM in SPSS) was used to assess the unadjusted and adjusted differences in the QOLS data prior to fracture between groups (wrist fracture patients versus controls and hip fracture patients versus controls). The QOLS score was transformed to Z-scores when used as a dependent variable in the multiple regression analysis. Independent variables were entered in a block-wise manner; demographic variables (age, sex, education level, and marital status) were entered in the first block, clinical fracture variables (osteoporosis, falls, and fracture groups or controls) were entered in the second block, and finally health-focused QOL (SF-36 PCS and SF-36 MCS) scores were entered. The unstandardized regression coefficients were used as effect parameters, and, because the Z-scores were used as dependent variables, these coefficients may be interpreted as standard difference scores (S-scores); i.e., they allow for comparisons of effect sizes across different independent variables in the unadjusted and adjusted analyses. The values of the regression coefficients were interpreted according to Cohen's effect size index, in which coefficients in the range 0.2–0.5 are defined as indicating a small difference, 0.5–0.8 a moderate difference, and 0.8 or more a large difference [47]. In the final regression analyses, we also transformed PCS and MCS to Z-scores.

Interactions between pairs of independent variables were tested, one pair at a time. The level of significance was set at 0.05.

## Results

### Patients and the control group

Differences in demographics and clinical characteristics prior to fracture between the study groups are shown in Table 1. The hip fracture patients were on average eight years older than both the wrist fracture patients and the controls (p = 0.003). The excluded wrist patients (mean age, 76.0 ± 11.5 years) on average were nine years older than the wrist patients accepted into the study (p < 0.001). The wrist patients who were unwilling to participate (mean age, 71.8 ± 11.2 years) on average were five years older than the participants (p < 0.001). The excluded hip patients (mean age, 84.0 ± 8.0 years), on average were nine years older than the patients in the study (p < 0.001), and the hip patients who were unwilling to participate (mean age, 81.0 ± 8.0 years), were six years older than the included patients (p < 0.001).

**Table 1:** Demographics and clinical variables in the wrist fracture patients, hip fracture patients, and the control group.

	**Wrist**	**Hip**	**Control group**	p value*
	n = 181	n = 97	n = 226	
**Demographics**				
Age (years)	66.9 (9.9)	74.9 (9.8)	66.8 (9.0)	**0.003 bc**
Females	161 (89)	71 (73)	192 (85)	**0.003 bc**
BMI (kg/m^2^)	25.4 (4.3)	23.1 (4.0)	26.6 (4.3)	**< 0.001 abc**
Education level				**0.040 b**
< 10 years	62 (37)	40 (48)	88 (39)	
11–13 years	70 (42)	29 (35)	70 (31)	
> 13 years	36 (21)	14 (17)	67 (30)	
Cohabiting	92 (53)	34 (36)	152 (68)	**< 0.001 abc**
Regular exercise**	134 (74)	57 (59)	169 (77)	**0.008 bc**
Current smoker	29 (16)	29 (30)	30 (13)	**0.001 bc**
**Clinical characteristics**				
Current glucocorticoids	12 (7)	12 (13)	3 (1)	**< 0.001 ab**
Current calcium and/or vitamin D	40 (22)	20 (21)	50 (22)	0.950
Current ART	26 (14)	9 (9)	13 (6)	**0.013 a**
Previous fracture(s)	97(54)	46 (48)	97 (45)	0.175
≥ 1 fall in previous year	75 (47)	48 (53)	67 (36)	0.023b
Osteoporosis***	60 (33)	57 (59)	37 (16)	**< 0.001 abc**
**Health-focused QOL (SF-36)**				
SF-36 PCS****	50.9 (9.8)	46.3 (10.5)	51.3 (8.3)	**< 0.001 bc**
SF-36 MCS****	50.1 (9.9)	47.7 (11.6)	50.7 (8.7)	**0.044 b**

Continuous variables are presented as mean and standard deviation (SD), and group variables as numbers and per cent (%).

* Chi-square used to compare categorical data, and ANOVA with Bonferroni post hoc test used for continuous variables. Significant differences between the marked groups: a = wrist fracture patients vs. control group, b = hip fracture patient vs. control group, and c = wrist fracture patients vs. hip fracture patients. P-values marked with bold indicate statistically significant differences between the groups.

** Exercise more than 30 minutes three times a week.

*** Osteoporosis at total hip and/or spine L2–L4.

**** SF-36 scores range from 0 to 100, where 100 means perfect health.

Specific osteoporosis treatment: oestrogens, biphosponates, or selective oestrogen receptor modulators.

ART, antiresorptive treatment; BMI, body mass index; PCS, physical component summary; MCS, mental component summary.

Both the wrist and the hip fracture patients had significantly lower BMI than the controls (p < 0.001). The hip fracture patients had less years of education than the controls (p = 0.006). Compared with the controls and the wrist fracture patients, the hip fracture patients exercised less (p = 0.008), tended to fall more often (p = 0.023), and were more likely to smoke (p = 0.001). Osteoporosis at one or both of the total hip or lumbar spine L2–L4 was found in 33% of the wrist fracture patients, 59% of the hip fracture patients, and 16% of the controls. The difference in frequency of osteoporosis between the three groups was significant (Table 1).

The correlation between the overall QOLS score and PCS prior to fracture was r = 0.42 (p < 0.001) and between QOLS and MCS, r = 0.58 (p < 0.001) in the entire study population. The hip fracture patients reported a significantly lower PCS score than both the control group and the wrist fracture patients (p < 0.001). The MCS score was significantly lower in the hip fracture patients than in the control group (p = 0.040). Some of these differences between the hip patients and controls might be related to the older age of the hip patients.

Co-morbidities such as heart diseases (p = 0.002), lung diseases (p = 0.036), and urogenital diseases (p = 0.003) were reported significantly more frequently by the hip fracture patients than by both the wrist fracture patients and the controls. Menopause status and mean age at menopause did not differ between the female fracture patients and controls.

### Unadjusted differences in GQOL between the fracture patients and controls prior to fracture

The wrist fracture patients and the control group reported significantly higher total QOLS scores than the hip fracture patients (p < 0.001). The same pattern was seen for the two sub-dimensions of QOLS: relationship and marital well-being, and health and functioning (both p < 0.001). Scores for personal, social, and community commitment were significantly lower in the hip fracture patients than in the controls (p = 0.004). The GQOL scores did not differ significantly between the wrist patients and the controls (Table 2).

**Table 2:** QOLS scores for relationship and marital well-being, health and functioning, and personal, social, and community commitment in wrist fracture patients, hip fracture patients, and controls.

	**Wrist fracture patients**	**Hip fracture patients**	**Control group**	p value*
	n = 181	n = 97	n = 226	

**QOLS**				
Total QOLS**	94.03 (10.65)	89.29 (10.98)	95.97 (9.20)	**< 0.001 bc**
Relationship and marital well-being***	31.24 (3.07)	29.67 (3.70)	31.75 (2.88)	**< 0.001 bc**
Health and functioning***	28.81 (4.15)	26.61 (5.09)	29.54 (3.77)	**<0.001 bc**
Personal, social, and community commitment****	33.97 (5.00)	32.70 (4.74)	34.57 (4.33)	**0.006 b**

Unadjusted means (SD)

Values are expressed as mean (SD).

* UNIANOVA. Significant differences between: a = wrist vs. control, b = hip vs. control, and c = wrist vs. hip. P-values marked with bold indicate statistically significant differences between the groups.

**The QOLS scores range from 16 to 112, where 112 means perfect QOL.

***Range 5–35, where 35 means high QOL.

****Range 6–42, where 42 means high QOL.

The effects sizes were moderate for the unadjusted differences in QOLS between the hip fracture group and the control group (S-score = -0.62), relationship and marital well-being (S-score = -0.64), and health and functioning (S-score = -0.62).

### Adjusted differences in GQOL between the fracture patients and controls prior to fracture

**Figure 1:**
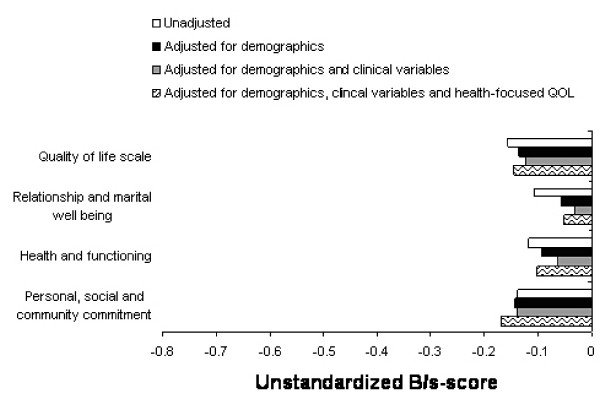
Differences between the controls and wrist fracture patients in unstandardized B/S-scores using multiple regression analysis to adjust the blocks of independent variables.

Adjusting for demographics, clinical fracture variables, and health-focused QOL in the QOLS and the three sub-dimensions produced no significant differences between the wrist patients and the controls (Figure 1).

**Figure 2:**
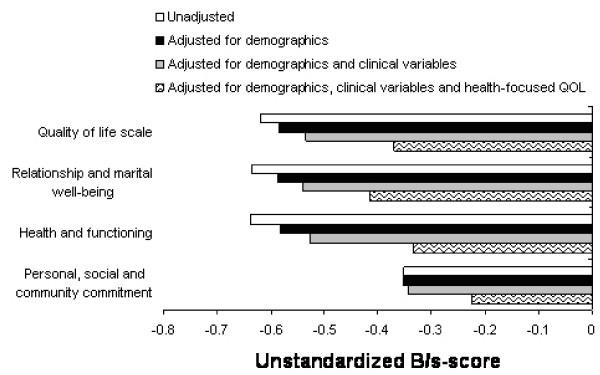
Differences between the controls and hip fracture patients in unstandardized B/S-scores using multiple regression analysis to adjust the blocks of independent variables.

Adjusting for demographic and clinical fracture variables decreased the differences in QOLS between the hip patients and control groups, but the differences were still significant (p < 0.001) (Figure 2). Adjusting for all demographics, clinical fracture variables, and health-focused QOL reduced the differences between groups substantially more (p = 0.001). The independent variables explained 51.4% of the variance in QOLS (Table 3).

**Table 3:** Regression analysis of demographics, clinical characteristics, and health status on QOLS and its sub-dimensions (transformed to Z-scores).

	**Quality of life scale**			**Relationship and marital well-being**			**Health and functioning**			**Personal, social, and community commitment**		
	**Adjusted B**	**95% CI**	**p value**	**Adjusted B**	**95% CI**	**p value**	**Adjusted B**	**95% CI**	**p value**	**Adjusted B**	**95% CI**	**p value**

**Demographic**												
Age*	0.14	(0.06, 0.23)	**0.001**	0.14	(0.04, 0.24)	**0.008**	0.12	(0.04, 0.20)	**0.002**	0.10	(0.00, 0.21)	0.051
Sex	0.30	(0.09, 0.51)	**0.005**	0.21	(-0.04, 0.45)	0.093	0.21	(0.02, 0.41)	**0.028**	0.31	(0.05, 0.56)	**0.019**
Education												
< 10 yr												
11–13 yr	0.20	(0.03, 0.37)	**0.021**	0.18	(-0.02, 0.38)	0.075	0.19	(0.04, 0.34)	**0.014**	0.13	(-0.08, 0.33)	0.230
> 13 yr	0.13	(-0.07, 0.32)	0.211	0.07	(-0.17, 0.30)	0.566	0.11	(-0.08, 0.29)	0.251	0.13	(-0.12, 0.37)	0.308
Marital status	-0.16	(-0.32, -0.003)	**0.045**	-0.35	(-0.53, -0.16)	**< 0.001**	-0.12	(-0.26, 0.03)	0.113	-0.003	(-0.20, 0.19)	0.975
**Clinical**												
Wrist fracture	-0.15	(-0.31, 0.02)	0.077	-0.05	(-0.24, 0.14)	0.592	-0.10	(-0.25, 0.05)	0.178	-0.17	(-0.37, 0.03)	0.099
Hip fracture	-0.37	(-0.59, -0.15)	**0.001**	-0.41	(-0.67, -0.16)	**0.002**	-0.33	(-0.53, -0.14)	**0.001**	-0.23	(-0.49, 0.04)	0.096
Osteoporosis**	0.003	(-0.17, 0.18)	0.975	0.02	(-0.19, 0.22)	0.872	0.04	(-0.12, 0.19)	0.635	0.004	(-0.21, 0.22)	0.967
≥ 1 fall in the last year	0.01	(-0.14, 0.16)	0.908	0.10	(-0.08, 0.27)	0.275	0.04	(-0.09, 0.18)	0.542	-0.09	(-0.27, 0.09)	0.342
**Health-focused QOL**												
ZPCS***	0.38	(0.30, 0.45)	**< 0.001**	0.22	(0.13, 0.31)	**< 0.001**	0.48	(0.41, 0.55)	**< 0.001**	0.25	(0.16, 0.35)	**< 0.001**
ZMCS***	0.51	(0.44, 0.59)	**< 0.001**	0.45	(0.37, 0.54)	**< 0.001**	0.49	(0.42, 0.56)	**< 0.001**	0.39	(0.30, 0.47)	**< 0.001**
**R**^2^**adjusted**	51.4%			35.2%			59.3%			24.9%		

Adjusted unstandardized regression coefficients, 95% CI, p values, and multiple R^2 ^for the full model.

P-values marked with bold indicate statistically significant p-values.

* Age in decades.

** Osteoporosis at total hip and/or spine L2–L4.

*** ZPCS, physical component summary transformed to Z-score; ZMCS, mental component summary transformed to Z-score.

After adjusting for all demographics, clinical fracture variables, and health-focused QOL for the sub-dimension of relationship and marital well-being, the differences between the hip patients and controls remained significant (p = 0.002) (Figure 2). The variables in the full model explained 35.2% of the variance in relationship and marital well-being (Table 3). Adjusting for demographics and clinical fracture variables in the sub-dimension of health and functioning reduced the differences between groups (p < 0.001) (Figure 2). After adjusting for all demographics, clinical fracture variables, and health-focused QOL in the dimension of health and functioning, the differences between the hip patients and controls remained significant (p = 0.001). The independent variables in the full model explained 59.3% of the variance in health and functioning (Table 3), and most of the variance was explained by the association with health-focused QOL measured in the SF-36.

After adjusting all demographic, clinical fracture variables, and health-focused QOL for the sub-dimension of personal, social, and community commitment, the differences between the hip patients and controls were not significant (Table 3). The independent variables explained 24.9% of the variance in personal, social, and community commitment.

**Figure 3:**
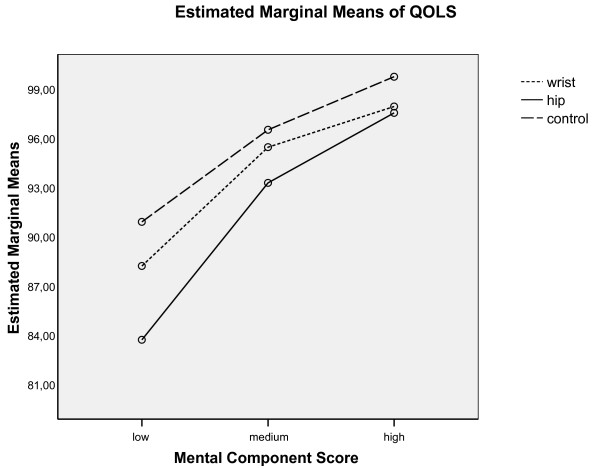
Interaction between MCS and patient group or control group.

Differences in QOL scores between comparison groups were particularly pronounced at the lowest levels (tertile) of MCS. The adjusted mean QOLS score was 69.5 in hip patients, 74.2 in wrist patients, and 77.0 in controls. Differences in QOLS between groups were substantially smaller at higher levels of MCS (Figure 3).

## Discussion

This is the first study to assess GQOL in patients with low-energy wrist and hip fractures and to compare the scores with age-and sex-matched controls. The hip fracture patients reported lower GQOL before the fracture occurred compared with controls. Adjusting for known covariates of GQOL decreased these differences substantially, but the differences between the hip fracture group and controls remained significant. However, unadjusted and adjusted GQOL scores before the fracture did not differ between the wrist patients and controls.

Adjusting for well-known predictors of QOLS such as age, sex, education level, marital status, clinical characteristic, and health-focused QOL reduced the differences between the hip patients and controls in our study, but the differences remained significant [48-56]. We expected these adjustments to eliminate or reduce the differences between the hip patients and controls substantially more than what we found. The remaining differences might be explained by more co-morbidity and lower physical function caused by aging and age-related diseases in the hip group, which were not captured by the SF-36.

The variance in QOLS and the three sub-dimensions explained by health-focused QOL was substantial, especially the mental component. A strong association between mental health and GQOL has been reported by others [53,55-59]. In a meta-analysis of the QOL literature that distinguished between QOL and health status, Smith at al. [57] found that patients give greater emphasis to mental health than physical functioning when rating GQOL. Our findings seem to be consistent with the meta-analysis by Smith at al. [57].

Wilson and Cleary [20] proposed a model to classify different measures of health outcomes. They divided the outcomes on a continuum comprising five levels: biological and physiological factors, symptoms, functioning, general health perception, and overall QOL. Patients' preferences and emotional or psychological factors play important roles at several points in the model and are particularly important in understanding general health perceptions and GQOL. In addition, perceptions of health appear to be more important than objective health in terms of their effects on GQOL [49]. Although we did not include measures of patients' preferences and emotional factors in our analysis, our data seem to coincide with the pattern described by Wilson and Cleary. The associations proposed in their model may explain the strong correlation between the health-focused QOL and GQOL and the weak correlation between clinical fracture characteristics and GQOL in our study. Both Osoba [18] and Ferrans et al [17] present adjusted Wilson and Cleary [20] models, emphasizing the bidirectional relationship between health- focused QOL and GQOL (and the other health outcomes in the model), which is also seen in our study. However health-focused and global-QOL are distinct as health-focused QOL centres on the individual's experience of general state of health, such as physical, social, and mental well-being, while GQOL focuses on the individual's satisfaction with life as a whole [17,18,60].

Our study has some limitations, which should be considered when interpreting the findings. The patients were asked to evaluate their "pre-fracture" GQOL after the fracture had occurred. Changes in health, such as experiencing a fracture, might cause a shift in how the patients judged their GQOL (selective reporting bias and response shift) [61]. On the other hand, patients who have experienced a recent change in health are more likely to make accurate responses [5,16]. Furthermore, have a short time span since events shown be important to report more accurate QOL [62,63]. The questionnaire was designed with a clear instruction that the patients should think of the period before the fracture, and in most of the patients, GQOL was assessed within the first two weeks after the fracture. It seems unlikely that the patients were unable to recall their GQOL immediately before and at the time of the fracture. Furthermore, the method used to in our study to assess GQOL the week before fracture, seems to be the most realistic and appropriate alternative.

The patients were asked to describe their GQOL at the time before the fracture, whilst health-focused QOL was more specifically restricted to the 4 weeks before the fracture [21,35,43,60]. The restricted time span with regard to health-focused QOL assessment could raise doubts regarding, the prudence of measuring GQOL and health-focused QOL within the same time before the fracture. Studies have shown that patients tend to think of the time before the event regardless of the instructions specifying "the time before" the event (fracture) or "the four weeks before" the event (fracture) [63-65]. Furthermore, both questionnaires were followed by the instruction to relate to the time before the fracture occurred [16,62,63].

We chose to use imputation techniques with regard to missing values in the QOLS questionnaire when at least 80% of the items had valid response. Some doubts have been raised regarding this technique, because of the underlying assumptions. However, it should be emphasized that failing to impute missing data also involves making assumptions and may have negative consequences. Patients failing to respond one or more items are then deleted as non-responders in furthur analyses, thereby reducing statistical power and possibly biasing the sample being analyzed [16].

All patients included in the study were identified at the hospital, which is the only referral centre for orthopaedic trauma in the region. Hence, the external validity of the study should be satisfactory. A high number of the hip fracture patients (n = 271) did not fulfil the inclusion criteria. Closer examination showed that most of these patients were nursing home residents who suffered from dementia, confusion, or severe diseases, and they were older than the participants. Hence, it is likely that the excluded hip fracture patients had more impaired health than those included in the present study and that the results for the hip fracture patients may be generalized only to people residing in their own homes. The patients unwilling to participate in the study were older than the participants were. Younger patients might be more aware of the benefits of participating in a study like this. The older age of the patients who were unwilling to participate might also be related to aging and age-related diseases in this group, and we probably reached the most healthy fracture patients [66].

The findings in our study are based on fewer participants less in the hip group than in the wrist group, and hip patients are slightly older than wrist patients. Even thought both wrist and hip fractures are strongly associated with objective health factors like osteoporosis and falls, we found that wrist and hip fracture patients are quite different with regard to demographics and clinical variables. However, when comparing wrist fracture patients versus controls and hip fracture patients versus controls with regard to GQOL, known covariates of GQOL like age, sex, education, marital status, clinical variables and health-focused QOL were adjusted for in the multivariate analysis. Such adjustments allows for a more meaningful comparison of GQOL between fracture patients and controls by removing the possible effects of "confounders" (common underlying causes) of GQOL and group membership [42]. Rather than aiming for a study population with "balanced" comparison groups with the same number of participants in each, we included all eligible participants, thus decreasing confidence intervals and increasing statistical power [42].

Hip fracture patients had a lower GQOL even before the fracture occurred, and they seemed to be less satisfied with life as a whole. GQOL assessment seems to add knowledge to the complexity of the conditions prior to fracture, and decreased GQOL in elderly seem to be an independent associate of low energy hip fracture. Decreased GQOL have been identified as an associate of other diseases and conditions as well [56]. However, our findings suggest that by identifying patients with low GQOL, in addition to other known risk factors for hip fracture, may rise the probability to target preventive health care activities. Preventive programmes might include efforts to help reduce the tendency to fall, improve the patient's diet and help him or her stop smoking, increase physical activity [2], and promote better GQOL.

It is unknown how low GQOL before a fracture occurs influences rehabilitation after the fracture, and prospective studies are needed to answer this question. This knowledge would help healthcare providers develop and initiate prevention and rehabilitation efforts.

## Conclusion

This is the first study to compare GQOL in patients with a low-energy wrist fracture or hip fracture with GQOL scores in matched controls. The hip fracture patients reported lower GQOL before the fracture, even after adjusting for known predictors of GQOL. The current state of research may leave opportunities to identify groups of individuals who might benefit from prevention efforts.

## Abbreviations

BMD: bone mineral density; BMI: body mass index; DXA: dual-energy X-ray, absorptiometry; GQOL: global quality of life; MCS: mental component summary; PCS: physical component summary; SF-36: Short Form-36; QOL: quality of life; QOLS: Quality of Life Scale; WHO: World Health Organization

## Competing interests

The authors declare that they have no competing interests.

## Authors' contributions

GR initiated this paper as a part of a larger study of fracture patients, collected and analyzed the data and wrote the manuscript. GH was the principal investigator for the research program in patients with low energy wrist and hip fracture. AM supervised GR during the analyzes and drafting of the paper. TM provided statistical advice. AKW supervised GR during the analyzes and drafting of the paper. All authors critiqued revisions of the paper and approved the final manuscript
